# 3-(4-Fluoro­phen­yl)imidazo[1,2-*a*]pyridine-2-carbaldehyde

**DOI:** 10.1107/S241431462500553X

**Published:** 2025-06-24

**Authors:** Firudin I. Guseinov, Aida I. Samigullina, Tuncer Hökelek, Sahil Z. Hamidov, Jamal Lasri, Khudayar I. Hasanov, Narmina A. Guliyeva, Alebel N. Belay

**Affiliations:** a Kosygin State University of Russia, 117997 Moscow, Russian Federation; b N. D. Zelinsky Institute of Organic Chemistry, Russian Academy of Sciences, 119991 Moscow, Russian Federation; c Hacettepe University, Department of Physics, 06800 Beytepe-Ankara, Türkiye; d Azerbaijan Technological University, Khatai Avenue, Ganja 2011, Azerbaijan; e Department of Chemistry Rabigh College of Science and Arts King Abdulaziz University,Jeddah 21589 Saudi Arabia; f Azerbaijan Medical University, Scientific Research Centre (SCR), A. Kasumzade St. 14, AZ1022 Baku, Azerbaijan; g Department of Chemical Engineering, Baku Engineering University, Hasan Aliyev str. 120, Baku, Absheron AZ0101, Azerbaijan; h Department of Chemistry, Bahir Dar University, PO Box 79, Bahir Dar, Ethiopia; University of Aberdeen, United Kingdom

**Keywords:** crystal structure, hydrogen bond, Hirshfeld surface

## Abstract

In the title compound, C_14_H_9_FN_2_O, the dihedral angle between the imidazo–pyridine fused ring system and the pendant fluoro­phenyl ring is 53.77 (4)°. In the crystal, C—H⋯O and C—H⋯F hydrogen bonds link the mol­ecules into a three-dimensional network. A Hirshfeld surface analysis of the crystal structure indicates that the most important contributions for the crystal packing are from H⋯H (30.4%), H⋯C/C⋯H (23.7%), H⋯O/O⋯H (12.2%) and H⋯F/F⋯H (11.1%) inter­actions.

## Abstract 2

In the title compound, C—H⋯O and C—H⋯F hydrogen bonds link the mol­ecules into a three-dimensional network.

## Graphical abstract

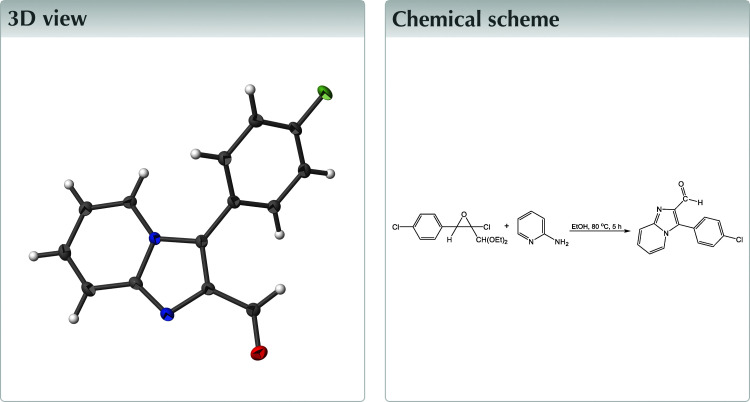

## Structure description

**Figure 1:**
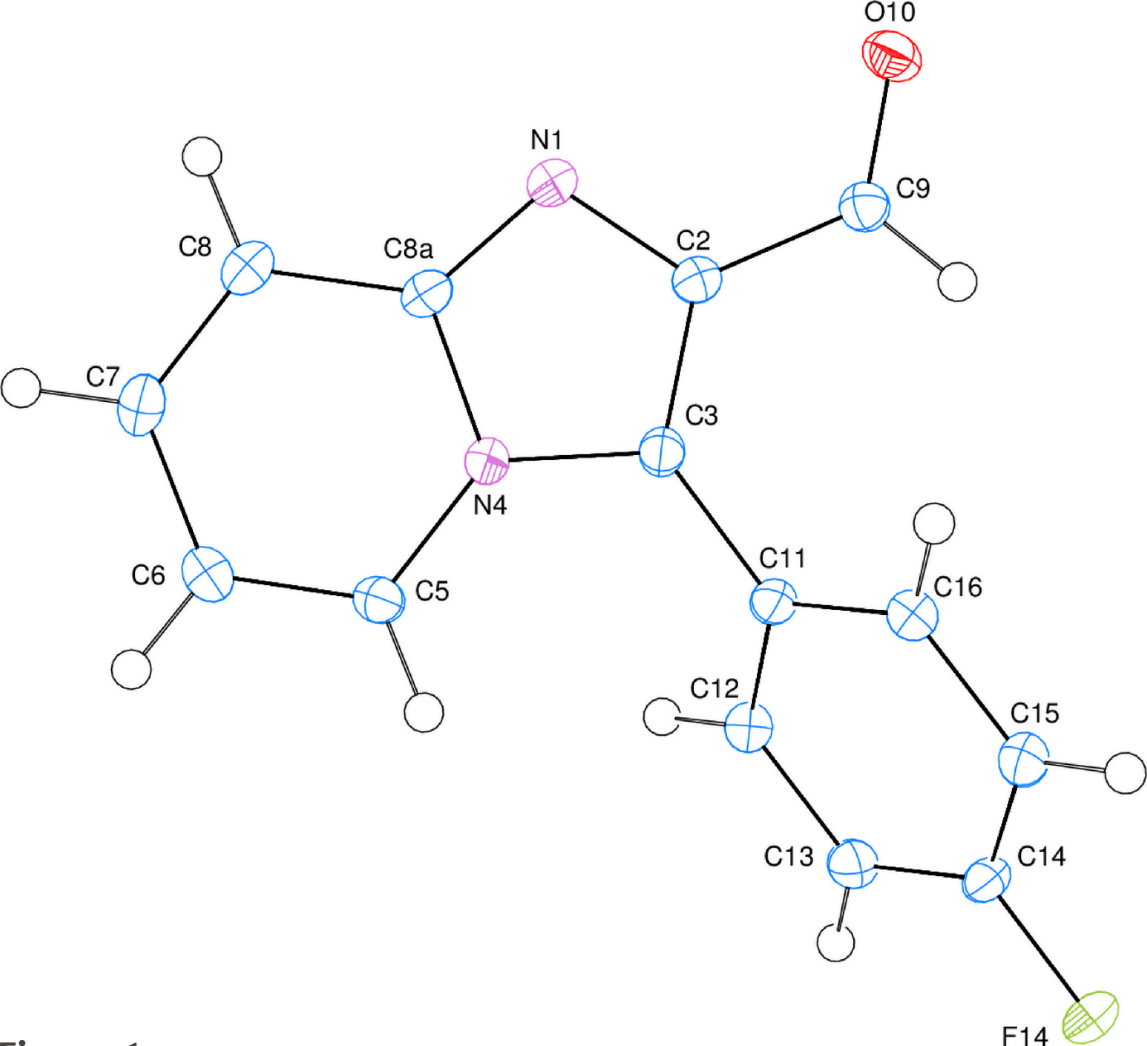
The mol­ecular structure of (**I**) showing 50% probability displacement ellipsoids.

Imidazo[1,2-*a*]pyridines are an important class of fused N-bridged compounds because of the broad spectrum of synthetic transformations as well as biological activity profiles displayed, which are strongly affected by the substitutions. Several imidazo[1,2-*a*]pyridines are used clinically, such as the unsubstituted imidazole skeleton cardiotonic agent olprinone, the anti­cancer agent zolimidine, the 2-substituted analgesic miroprofen, the 3-substituted anti­osteoporosis 2,3-disubstituted derivatives with anxiolytic and sedative properties, saripidem, alpidem, and necopidem, and the agent for the treatment of brain disorders and insomnia, zolpidem (Kurteva, 2021 ▸). As part of our ongoing studies in this area, we now report the synthesis and structure of the title compound (**I**) (Fig. 1 ▸).

The dihedral angle between the N1/N2/C2/C3/C5–C8/C8*A* fused ring system (r.m.s. deviation = 0.015 Å) and the pendant C11–C16 fluoro­benzene ring is 53.77 (4)°. The aldehyde O atom lies close to the plane of the fused ring as indicted by the N1—C2—C9=O10 torsion angle of 4.15 (17)°. Atom F14 lies −0.0118 (8) Å away from the best least-squares plane of the phenyl ring.

**Figure 2:**
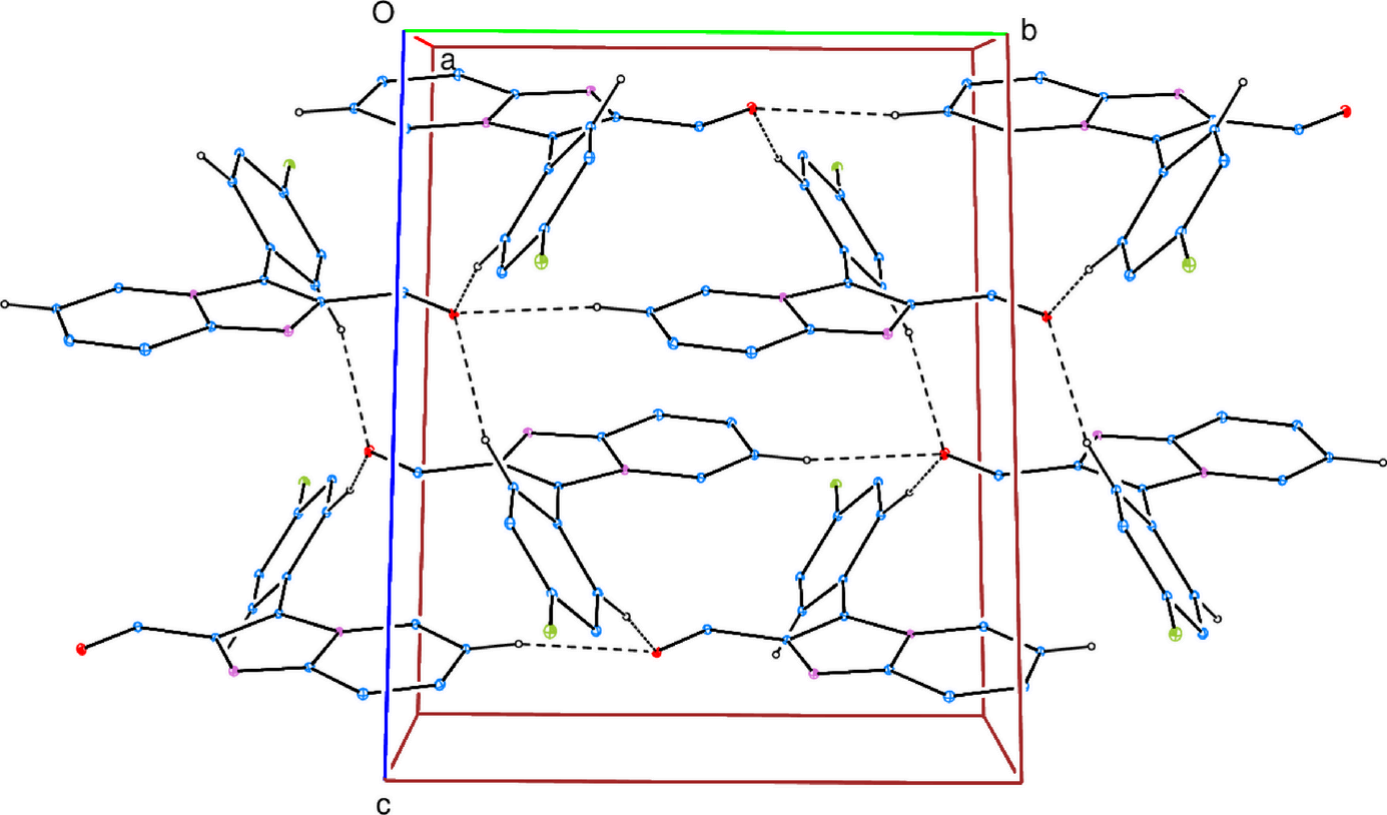
A partial packing diagram of (**I**) viewed down the *a*-axis direction with C—H⋯O hydrogen bonds shown as dashed lines. Hydrogen atoms not involved in these inter­actions have been omitted for clarity.

In the crystal, C—H⋯O and C—H⋯F hydrogen bonds (Table 1 ▸) link the mol­ecules into a three-dimensional network, enclosing 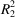
(14), 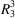
(16), 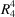
(18), 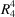
(24) and 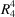
(30) ring motifs (Fig. 2 ▸). It may be noted that the aldehyde O10 atom accepts three such bonds in a distorted tetra­hedral arrangement (including the C8=O10 bond). Atom F14 accepts two hydrogen bonds in a distorted trigonal arrangement including the C14—F1 bond. Further, there is a weak π–π stacking inter­action between the pyridine rings with a centroid–centroid distance of 3.9090 (7) Å. No significant C—H⋯π inter­actions are observed.

**Figure 3:**
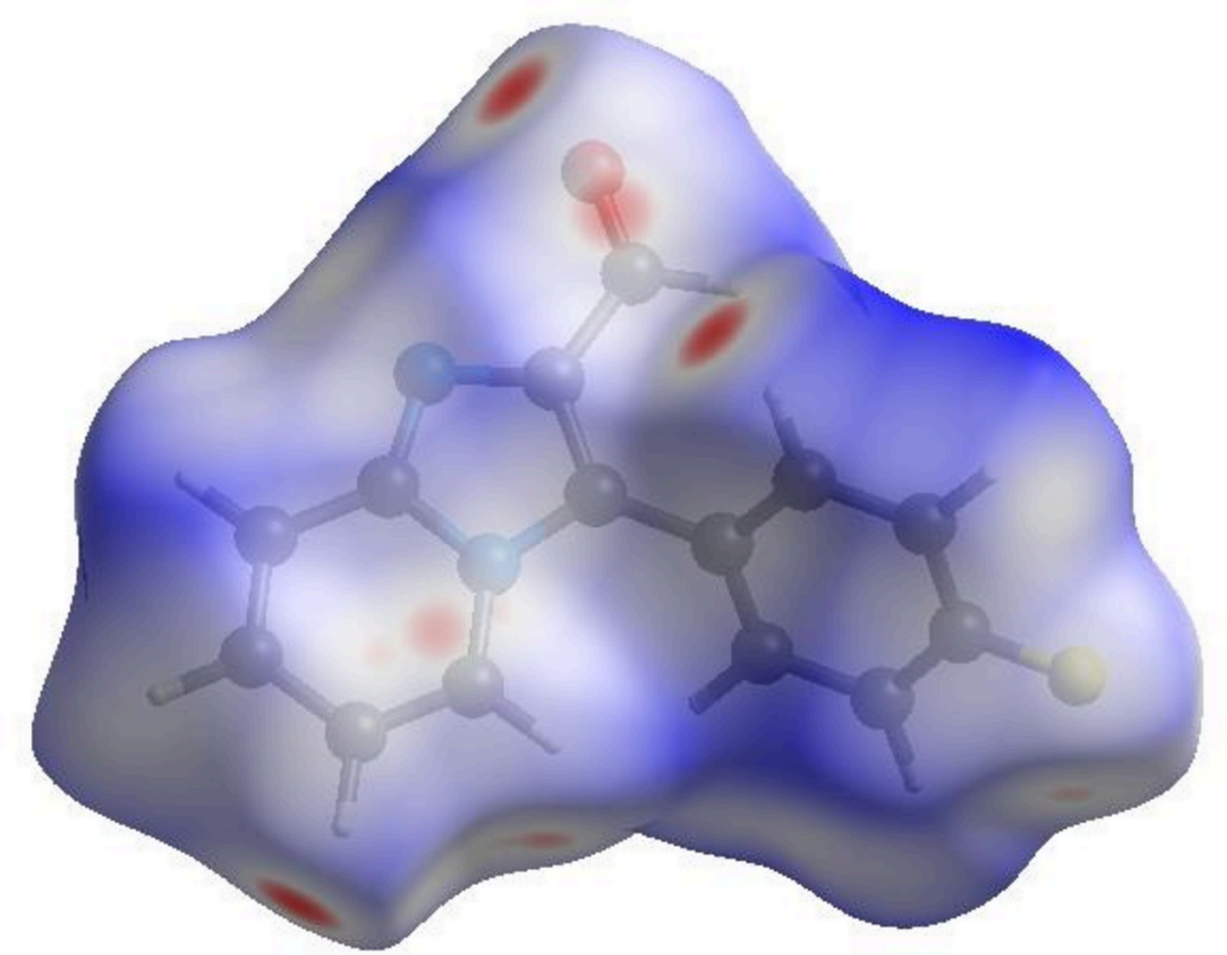
View of the three-dimensional Hirshfeld surface of (**I**) plotted over *d*_norm_.

**Figure 4:**
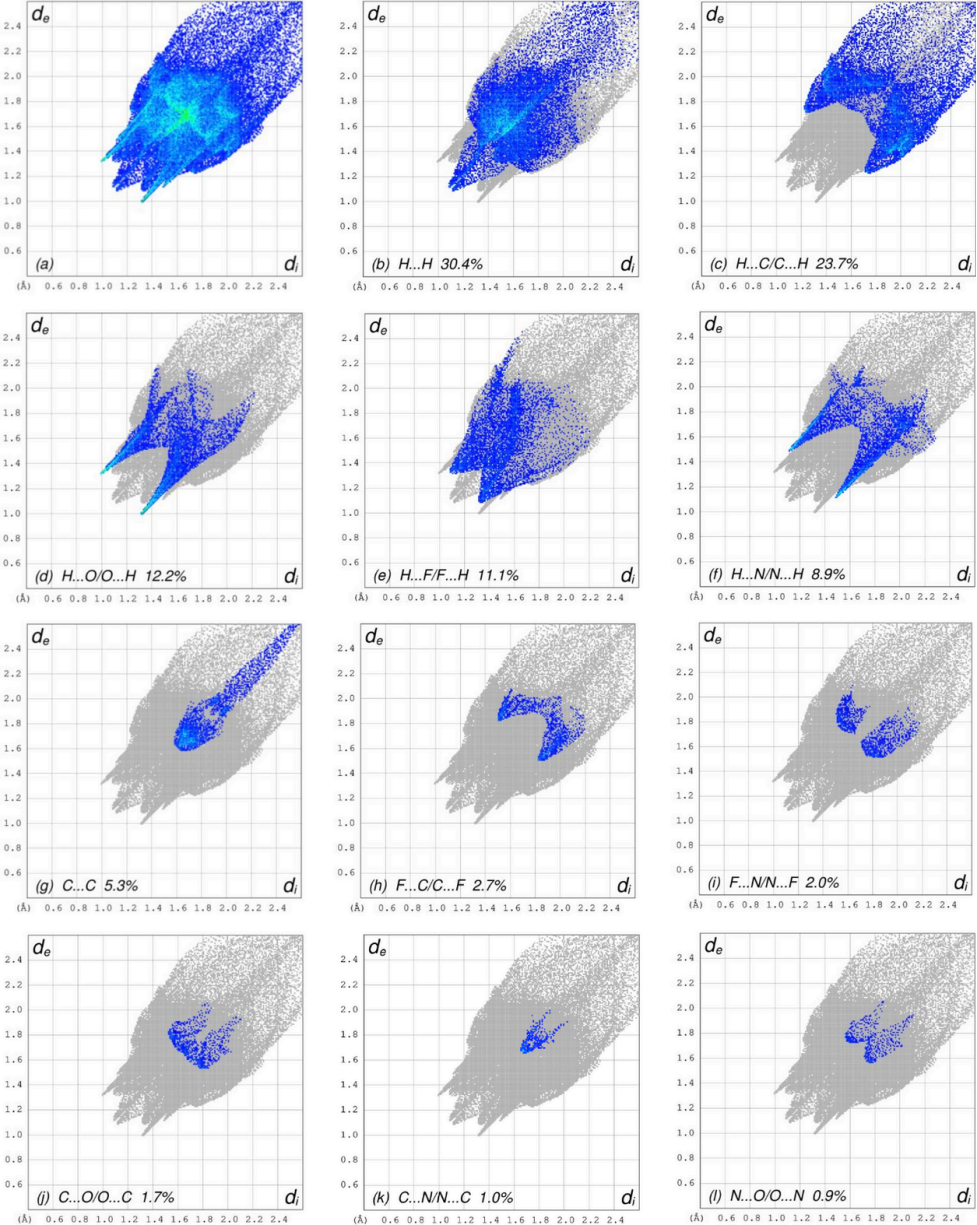
The two-dimensional fingerprint plots for (**I**), showing (*a*) all inter­actions, and delineated into (*b*) H⋯H, (*c*) H⋯C/C⋯H, (*d*) H⋯O/O⋯H, (*e*) H⋯F/F⋯H, (*f*) H⋯N/N⋯H, (*g*) C⋯C, (*h*) F⋯C/C⋯F, (*i*) F⋯N/N⋯F, (*j*) C⋯O/O⋯C, (*k*) C⋯N/N⋯C and (*l*) N⋯O/O⋯N inter­actions. The *d*_i_ and *d*_e_ values are the closest inter­nal and external distances (in Å) from given points on the Hirshfeld surface.

In order to further visualize the inter­molecular inter­actions in the crystal of (**I**), a Hirshfeld surface analysis (Fig. 3 ▸) was carried out using *Crystal Explorer 17.5* (Spackman *et al.*, 2021 ▸). The overall two-dimensional fingerprint plot, Fig. 4 ▸*a*, and those delineated into different contact types (McKinnon *et al.*, 2007 ▸) are illustrated in Fig. 4 ▸*b–l*. These indicate that the most important contributions to the crystal packing are from H⋯H (30.4%), H⋯C/C⋯H (23.7%), H⋯O/O⋯H (12.2%) and H⋯F/F⋯H (11.1%) inter­actions.

## Synthesis and crystallization

A solution of equimolar amounts of 2-amino­pyridine (2 mmol) and 2-chloro-2-(di­eth­oxy­meth­yl)-3-(4-fluoro­phen­yl)oxirane (2 mmol) in 20 ml of 95% aqueous ethanol was heated at reflux for 5 h. The solvent was removed *in vacuo*, and the remaining white powder was recrystallized from dry aceto­nitrile solution to give the title compound in the form of colorless prisms. Yield: 45%, m.p. 420–421 K. Analysis calculated for C_14_H_9_FN_2_O: C, 69.99; H, 3.78; N, 11.66. Found: C, 69.96; H, 3.75; N, 11.63. ^1^H NMR (300 MHz, DMSO-*d*_6_): 6.86–8.47 (8*H*, Ar) and 9.79 (1*H*, CHO). ^13^C NMR (200 MHz, DMSO-*d*_6_): 111.89, 115.78, 116.90, 111.87, 121.32, 126.18, 130.39, 132.79, 140.89, 147.48, 160.87, 165.96, 185.45.

## Refinement

Crystal data, data collection and structure refinement details are summarized in Table 2 ▸.

## Supplementary Material

Crystal structure: contains datablock(s) I. DOI: 10.1107/S241431462500553X/hb4525sup1.cif

Structure factors: contains datablock(s) I. DOI: 10.1107/S241431462500553X/hb4525Isup2.hkl

Supporting information file. DOI: 10.1107/S241431462500553X/hb4525Isup3.cml

CCDC reference: 2465675

Additional supporting information:  crystallographic information; 3D view; checkCIF report

Additional supporting information:  crystallographic information; 3D view; checkCIF report

## Figures and Tables

**Table 1:** Hydrogen-bond geometry (Å, °)

*D*—H⋯*A*	*D*—H	H⋯*A*	*D*⋯*A*	*D*—H⋯*A*
C6—H6⋯O10^i^	0.95	2.45	3.3579 (14)	160
C12—H12⋯O10^ii^	0.95	2.45	3.3449 (15)	158
C16—H16⋯O10^iii^	0.95	2.47	3.3754 (14)	159
C5—H5⋯F14^iv^	0.95	2.50	3.1435 (13)	125
C9—H9⋯F14^v^	0.95	2.55	3.2089 (14)	127

Symmetry codes: (i) 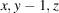; (ii) 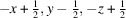; (iii) 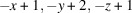; (iv) 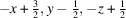; (v) 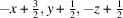.

**Table 2:** Experimental details

Crystal data	
Chemical formula	C_14_H_9_FN_2_O
*M*_r_	240.23
Crystal system, space group	Monoclinic, *P*2_1_/*n*
Temperature (K)	100
*a*, *b*, *c* (Å)	8.97833 (9), 10.13609 (9), 12.85096 (15)
β (°)	110.3575 (13)
*V* (Å^3^)	1096.46 (2)
*Z*	4
Radiation type	Cu *K*α
μ (mm^−1^)	0.88
Crystal size (mm)	0.39 × 0.30 × 0.16

Data collection	
Diffractometer	XtaLAB Synergy, Dualflex, HyPix
Absorption correction	Gaussian (*CrysAlis PRO*; Rigaku OD, 2023 ▸)
*T*_min_, *T*_max_	0.153, 1.000
No. of measured, independent and observed [*I* > 2σ(*I*)] reflections	13951, 2296, 2221
*R*_int_	0.023
(sin θ/λ)_max_ (Å^−1^)	0.632

Refinement	
*R*[*F*^2^ > 2σ(*F*^2^)], *wR*(*F*^2^), *S*	0.036, 0.096, 1.07
No. of reflections	2296
No. of parameters	164
H-atom treatment	H-atom parameters constrained
Δρ_max_, Δρ_min_ (e Å^−3^)	0.36, −0.17

Computer programs: *CrysAlis PRO* (Rigaku OD, 2023 ▸), *SHELXT2014/5* (Sheldrick, 2015*a* ▸), *SHELXL2018/3* (Sheldrick, 2015*b* ▸) and *OLEX2* (Dolomanov *et al.*, 2009 ▸).

## full crystallographic data

### 3-(4-Fluorophenyl)imidazo[1,2-a]pyridine-2-carbaldehyde Crystal data

**Table:**

C_14_H_9_FN_2_O	*F*(000) = 496
*M**_r_* = 240.23	*D*_x_ = 1.455 Mg m^−^^3^
Monoclinic, *P*2_1_/*n*	Cu *K*α radiation, λ = 1.54184 Å
*a* = 8.97833 (9) Å	Cell parameters from 10360 reflections
*b* = 10.13609 (9) Å	θ = 5.2–76.8°
*c* = 12.85096 (15) Å	µ = 0.88 mm^−^^1^
β = 110.3575 (13)°	*T* = 100 K
*V* = 1096.46 (2) Å^3^	Prism, colorless
*Z* = 4	0.39 × 0.30 × 0.16 mm

### 3-(4-Fluorophenyl)imidazo[1,2-a]pyridine-2-carbaldehyde Data collection

**Table:**

XtaLAB Synergy, Dualflex, HyPix diffractometer	2296 independent reflections
Radiation source: micro-focus sealed X-ray tube, PhotonJet (Cu) X-ray Source	2221 reflections with *I* > 2σ(*I*)
Mirror monochromator	*R*_int_ = 0.023
Detector resolution: 10.0000 pixels mm^-1^	θ_max_ = 77.0°, θ_min_ = 5.3°
ω scans	*h* = −10→11
Absorption correction: gaussian (CrysAlisPro; Rigaku OD, 2023)	*k* = −12→12
*T*_min_ = 0.153, *T*_max_ = 1.000	*l* = −16→15
13951 measured reflections	

### 3-(4-Fluorophenyl)imidazo[1,2-a]pyridine-2-carbaldehyde Refinement

**Table:**

Refinement on *F*^2^	Secondary atom site location: difference Fourier map
Least-squares matrix: full	Hydrogen site location: inferred from neighbouring sites
*R*[*F*^2^ > 2σ(*F*^2^)] = 0.036	H-atom parameters constrained
*wR*(*F*^2^) = 0.096	*w* = 1/[σ^2^(*F*_o_^2^) + (0.0493*P*)^2^ + 0.4295*P*] where *P* = (*F*_o_^2^ + 2*F*_c_^2^)/3
*S* = 1.07	(Δ/σ)_max_ = 0.001
2296 reflections	Δρ_max_ = 0.36 e Å^−^^3^
164 parameters	Δρ_min_ = −0.17 e Å^−^^3^
0 restraints	Extinction correction: *SHELXL2018/3* (Sheldrick, 2015b), Fc^*^=kFc[1+0.001xFc^2^λ^3^/sin(2θ)]^-1/4^
Primary atom site location: dual	Extinction coefficient: 0.0041 (4)

### 3-(4-Fluorophenyl)imidazo[1,2-a]pyridine-2-carbaldehyde Special details

**Table:**

Geometry. All esds (except the esd in the dihedral angle between two l.s. planes) are estimated using the full covariance matrix. The cell esds are taken into account individually in the estimation of esds in distances, angles and torsion angles; correlations between esds in cell parameters are only used when they are defined by crystal symmetry. An approximate (isotropic) treatment of cell esds is used for estimating esds involving l.s. planes.
Refinement. The C-bound hydrogen atom positions were calculated geometrically (C—H = 0.95 Å) and refined using a riding model by applying the constraint of *U*_iso_(H) = 1.2*U*_eq_(C).

### 3-(4-Fluorophenyl)imidazo[1,2-a]pyridine-2-carbaldehyde Fractional atomic coordinates and isotropic or equivalent isotropic displacement parameters (Å^2^)

**Table:**

	*x*	*y*	*z*	*U*_iso_*/*U*_eq_	
F14	0.94015 (9)	0.74538 (7)	0.18719 (7)	0.0262 (2)	
O10	0.32012 (9)	1.07889 (8)	0.39594 (7)	0.0216 (2)	
N4	0.37034 (10)	0.63485 (9)	0.37344 (7)	0.0149 (2)	
N1	0.24663 (11)	0.80627 (9)	0.42026 (8)	0.0175 (2)	
C3	0.44194 (13)	0.74722 (10)	0.35264 (9)	0.0156 (2)	
C2	0.36309 (12)	0.85016 (11)	0.38211 (8)	0.0157 (2)	
C11	0.57305 (13)	0.74542 (10)	0.30875 (10)	0.0165 (2)	
C8A	0.25290 (12)	0.67653 (11)	0.41485 (9)	0.0169 (2)	
C12	0.55893 (13)	0.67604 (11)	0.21145 (9)	0.0188 (2)	
H12	0.464190	0.628630	0.173733	0.023*	
C5	0.40337 (12)	0.50308 (10)	0.36430 (9)	0.0167 (2)	
H5	0.485900	0.478047	0.337956	0.020*	
C15	0.83753 (13)	0.81605 (11)	0.32299 (10)	0.0209 (3)	
H15	0.932676	0.863295	0.359970	0.025*	
C6	0.31690 (13)	0.41015 (11)	0.39326 (9)	0.0186 (2)	
H6	0.338214	0.319273	0.386936	0.022*	
C16	0.71280 (13)	0.81545 (10)	0.36391 (9)	0.0186 (2)	
H16	0.722589	0.862899	0.429719	0.022*	
C13	0.68258 (14)	0.67605 (11)	0.16965 (9)	0.0205 (2)	
H13	0.673737	0.629443	0.103622	0.025*	
C14	0.81886 (14)	0.74598 (10)	0.22711 (10)	0.0198 (3)	
C9	0.39053 (12)	0.99003 (11)	0.36905 (9)	0.0171 (2)	
H9	0.468252	1.013253	0.337566	0.021*	
C8	0.16225 (13)	0.57789 (12)	0.44436 (10)	0.0219 (3)	
H8	0.080367	0.602209	0.471573	0.026*	
C7	0.19366 (14)	0.44852 (12)	0.43332 (10)	0.0222 (3)	
H7	0.132621	0.382589	0.452579	0.027*	

### 3-(4-Fluorophenyl)imidazo[1,2-a]pyridine-2-carbaldehyde Atomic displacement parameters (Å^2^)

**Table:**

	*U*^11^	*U*^22^	*U*^33^	*U*^12^	*U*^13^	*U*^23^
F14	0.0238 (4)	0.0262 (4)	0.0375 (4)	−0.0010 (3)	0.0220 (3)	−0.0008 (3)
O10	0.0223 (4)	0.0171 (4)	0.0256 (4)	0.0027 (3)	0.0084 (3)	−0.0022 (3)
N4	0.0147 (4)	0.0141 (5)	0.0167 (4)	−0.0005 (3)	0.0068 (3)	0.0006 (3)
N1	0.0165 (4)	0.0179 (5)	0.0199 (4)	0.0003 (3)	0.0089 (4)	−0.0002 (3)
C3	0.0153 (5)	0.0149 (5)	0.0167 (5)	−0.0009 (4)	0.0060 (4)	0.0006 (4)
C2	0.0147 (5)	0.0167 (5)	0.0161 (5)	0.0005 (4)	0.0060 (4)	−0.0003 (4)
C11	0.0165 (5)	0.0143 (5)	0.0211 (5)	0.0022 (4)	0.0094 (4)	0.0030 (4)
C8A	0.0153 (5)	0.0194 (5)	0.0177 (5)	0.0007 (4)	0.0080 (4)	0.0001 (4)
C12	0.0191 (5)	0.0167 (5)	0.0224 (5)	−0.0007 (4)	0.0095 (4)	0.0003 (4)
C5	0.0174 (5)	0.0151 (5)	0.0180 (5)	0.0017 (4)	0.0066 (4)	−0.0005 (4)
C15	0.0177 (5)	0.0172 (5)	0.0296 (6)	−0.0009 (4)	0.0104 (4)	0.0004 (4)
C6	0.0210 (5)	0.0149 (5)	0.0189 (5)	−0.0001 (4)	0.0057 (4)	0.0002 (4)
C16	0.0191 (5)	0.0154 (5)	0.0232 (5)	0.0002 (4)	0.0096 (4)	−0.0004 (4)
C13	0.0249 (6)	0.0171 (5)	0.0237 (5)	0.0004 (4)	0.0138 (5)	−0.0003 (4)
C14	0.0192 (5)	0.0172 (5)	0.0292 (6)	0.0026 (4)	0.0162 (5)	0.0043 (4)
C9	0.0163 (5)	0.0167 (5)	0.0184 (5)	0.0004 (4)	0.0061 (4)	−0.0003 (4)
C8	0.0204 (5)	0.0232 (6)	0.0269 (6)	−0.0012 (4)	0.0142 (5)	0.0009 (4)
C7	0.0226 (5)	0.0210 (6)	0.0255 (6)	−0.0045 (4)	0.0116 (4)	0.0020 (4)

### 3-(4-Fluorophenyl)imidazo[1,2-a]pyridine-2-carbaldehyde Geometric parameters (Å, º)

**Table:**

F14—C14	1.3556 (13)	C5—H5	0.9500
O10—C9	1.2175 (14)	C5—C6	1.3527 (15)
N4—C3	1.3790 (13)	C15—H15	0.9500
N4—C8A	1.4013 (13)	C15—C16	1.3930 (15)
N4—C5	1.3820 (13)	C15—C14	1.3810 (17)
N1—C2	1.3741 (13)	C6—H6	0.9500
N1—C8A	1.3192 (15)	C6—C7	1.4272 (15)
C3—C2	1.3855 (15)	C16—H16	0.9500
C3—C11	1.4718 (14)	C13—H13	0.9500
C2—C9	1.4587 (15)	C13—C14	1.3843 (17)
C11—C12	1.4014 (15)	C9—H9	0.9500
C11—C16	1.4006 (15)	C8—H8	0.9500
C8A—C8	1.4215 (15)	C8—C7	1.3591 (17)
C12—H12	0.9500	C7—H7	0.9500
C12—C13	1.3914 (15)		
			
C3—N4—C8A	106.72 (9)	C14—C15—H15	121.0
C3—N4—C5	130.80 (9)	C14—C15—C16	118.08 (10)
C5—N4—C8A	122.39 (9)	C5—C6—H6	120.0
C8A—N1—C2	104.61 (9)	C5—C6—C7	120.05 (10)
N4—C3—C2	104.59 (9)	C7—C6—H6	120.0
N4—C3—C11	123.57 (9)	C11—C16—H16	119.8
C2—C3—C11	131.83 (9)	C15—C16—C11	120.49 (10)
N1—C2—C3	112.23 (10)	C15—C16—H16	119.8
N1—C2—C9	122.39 (10)	C12—C13—H13	121.0
C3—C2—C9	125.28 (10)	C14—C13—C12	118.03 (10)
C12—C11—C3	120.80 (10)	C14—C13—H13	121.0
C16—C11—C3	119.66 (10)	F14—C14—C15	118.41 (10)
C16—C11—C12	119.54 (10)	F14—C14—C13	118.24 (10)
N4—C8A—C8	117.75 (10)	C15—C14—C13	123.35 (10)
N1—C8A—N4	111.84 (9)	O10—C9—C2	124.14 (10)
N1—C8A—C8	130.41 (10)	O10—C9—H9	117.9
C11—C12—H12	119.7	C2—C9—H9	117.9
C13—C12—C11	120.52 (10)	C8A—C8—H8	120.3
C13—C12—H12	119.7	C7—C8—C8A	119.47 (10)
N4—C5—H5	120.4	C7—C8—H8	120.3
C6—C5—N4	119.27 (10)	C6—C7—H7	119.5
C6—C5—H5	120.4	C8—C7—C6	121.04 (10)
C16—C15—H15	121.0	C8—C7—H7	119.5
			
N4—C3—C2—N1	0.12 (12)	C11—C12—C13—C14	0.17 (16)
N4—C3—C2—C9	−176.34 (9)	C8A—N4—C3—C2	−0.30 (11)
N4—C3—C11—C12	53.26 (15)	C8A—N4—C3—C11	179.59 (10)
N4—C3—C11—C16	−127.70 (11)	C8A—N4—C5—C6	1.88 (16)
N4—C8A—C8—C7	1.04 (16)	C8A—N1—C2—C3	0.12 (12)
N4—C5—C6—C7	−0.33 (16)	C8A—N1—C2—C9	176.70 (10)
N1—C2—C9—O10	4.15 (17)	C8A—C8—C7—C6	0.41 (17)
N1—C8A—C8—C7	−178.51 (11)	C12—C11—C16—C15	−0.27 (16)
C3—N4—C8A—N1	0.41 (12)	C12—C13—C14—F14	179.47 (9)
C3—N4—C8A—C8	−179.23 (10)	C12—C13—C14—C15	−0.43 (17)
C3—N4—C5—C6	178.09 (10)	C5—N4—C3—C2	−176.96 (10)
C3—C2—C9—O10	−179.73 (10)	C5—N4—C3—C11	2.94 (18)
C3—C11—C12—C13	179.21 (10)	C5—N4—C8A—N1	177.41 (9)
C3—C11—C16—C15	−179.32 (10)	C5—N4—C8A—C8	−2.23 (15)
C2—N1—C8A—N4	−0.32 (11)	C5—C6—C7—C8	−0.80 (17)
C2—N1—C8A—C8	179.26 (11)	C16—C11—C12—C13	0.17 (16)
C2—C3—C11—C12	−126.88 (13)	C16—C15—C14—F14	−179.57 (9)
C2—C3—C11—C16	52.16 (17)	C16—C15—C14—C13	0.33 (17)
C11—C3—C2—N1	−179.76 (11)	C14—C15—C16—C11	0.03 (16)
C11—C3—C2—C9	3.8 (2)		

### 3-(4-Fluorophenyl)imidazo[1,2-a]pyridine-2-carbaldehyde Hydrogen-bond geometry (Å, º)

**Table:**

*D*—H···*A*	*D*—H	H···*A*	*D*···*A*	*D*—H···*A*
C6—H6···O10^i^	0.95	2.45	3.3579 (14)	160
C12—H12···O10^ii^	0.95	2.45	3.3449 (15)	158
C16—H16···O10^iii^	0.95	2.47	3.3754 (14)	159
C5—H5···F14^iv^	0.95	2.50	3.1435 (13)	125
C9—H9···F14^v^	0.95	2.55	3.2089 (14)	127

Symmetry codes: (i) *x*, *y*−1, *z*; (ii) −*x*+1/2, *y*−1/2, −*z*+1/2; (iii) −*x*+1, −*y*+2, −*z*+1; (iv) −*x*+3/2, *y*−1/2, −*z*+1/2; (v) −*x*+3/2, *y*+1/2, −*z*+1/2.
